# No Language-Specific Activation during Linguistic Processing of Observed Actions

**DOI:** 10.1371/journal.pone.0000891

**Published:** 2007-09-12

**Authors:** Ingo G. Meister, Marco Iacoboni

**Affiliations:** 1 Ahmanson-Lovelace Brain Mapping Center, David Geffen School of Medicine, University of California at Los Angeles, Los Angeles, California, United States of America; 2 Department of Psychiatry and Biobehavioral Sciences, David Geffen School of Medicine, University of California at Los Angeles, Los Angeles, California, United States of America; 3 Semel Institute for Neuroscience and Human Behavior, David Geffen School of Medicine, University of California at Los Angeles, Los Angeles, California, United States of America; 4 Brain Research Institute, David Geffen School of Medicine, University of California at Los Angeles, Los Angeles, California, United States of America; 5 Department of Neurology, University of Cologne, Cologne, Germany; 6 Max Planck Institute for Neurological Research with Klaus Joachim Zülch Laboratories of the Max Planck Society and the Medical Faculty of the University of Cologne, Cologne, Germany; University of Birmingham, United Kingdom

## Abstract

**Background:**

It has been suggested that cortical neural systems for language evolved from motor cortical systems, in particular from those fronto-parietal systems responding also to action observation. While previous studies have shown shared cortical systems for action – or action observation - and language, they did not address the question of whether linguistic processing of visual stimuli occurs only within a subset of fronto-parietal areas responding to action observation. If this is true, the hypothesis that language evolved from fronto-parietal systems matching action execution and action observation would be strongly reinforced.

**Methodology/ Principal Findings:**

We used functional magnetic resonance imaging (fMRI) while subjects watched video stimuli of hand-object-interactions and control photo stimuli of the objects and performed linguistic (conceptual and phonological), and perceptual tasks. Since stimuli were identical for linguistic and perceptual tasks, differential activations had to be related to task demands. The results revealed that the linguistic tasks activated left inferior frontal areas that were subsets of a large bilateral fronto-parietal network activated during action perception. Not a single cortical area demonstrated exclusive – or even simply higher - activation for the linguistic tasks compared to the action perception task.

**Conclusions:**

These results show that linguistic tasks do not only share common neural representations but essentially activate a subset of the action observation network if identical stimuli are used. Our findings strongly support the evolutionary hypothesis that fronto-parietal systems matching action execution and observation were co-opted for language, a process known as exaptation.

## Introduction

Since the initial observations on macaque mirror neurons - cells that fire while the monkey performs goal-directed actions and also while the animal observes somebody else's actions [1] - it has been speculated that these cells may have played a role in the evolution of language [2]–[4]. The theoretical arguments were substantially two: first, mirror neurons were originally discovered in a macaque brain area (area F5) that seems the homolog of human Broca's area, a major language center; second, mirror neurons seem to facilitate the *parity* between the sender and the receiver of a message, a parity that establishes what counts in communication [5], [6]. Recently, several labs have investigated shared neural systems between language and motor behavior in general, and language and premotor areas responding to action observation (thus, having mirroring properties) in particular. Taken together, the previous studies have demonstrated shared neural mechanisms - in the form of both activation maps [7]–[9] and modulation of neural excitability [10]–[13] - between the domain of language and of motor behavior in general, and action observation in particular. The extent to which neural systems for linguistic processing of visual stimuli is independent from the fronto-parietal mirror neuron system, however, has not been experimentally investigated so far (a completely different issue is obviously related to speech perception and superior temporal cortex: this issue is not investigated here). The experimental conditions of previous studies differed widely (e.g., motor tasks or action observation tasks on one side, and reading words or sentences on the other side), thus making the interpretation of differential activations between language and action (or action observation) quite difficult.

The present study adopted a design in which the experimental stimuli are identical, while task instructions differ, tapping either on linguistic functions or on action perception. By using such design, we believe we are in a position to test the extent to which linguistic processing of visual stimuli concerning actions and objects and human fronto-parietal areas responding to action observation overlap or differ. This question seems to us relevant to the hypothesis that mirror neurons played a key role in language evolution. Although evolutionary hypotheses cannot fully be demonstrated in the laboratory, we propose that the mirror neuron hypothesis of language evolution makes a relatively simple, and eminently tractable, prediction. If mirror neurons were initially selected for action observation (and presumably its understanding) and subsequently co-opted for language, - a process also known as exaptation [14] - one would expect that while processing identical visual stimuli, a linguistic task should activate a subset of or even all the areas activated by an action perception task, while no additional areas should be activated by the linguistic task. If additional areas are activated by the linguistic tasks, these additional areas should presumably be exclusively linguistic in nature and may not have evolved from mirror neurons.

## Methods

14 healthy right handed subjects, all of them native english speakers, were investigated (age 25.1±2.6 years, 5 men). The study was approved by the UCLA Institutional Review Board and all subjects gave written informed consent for participation in this study.

### Task and stimuli

**Figure 1:**
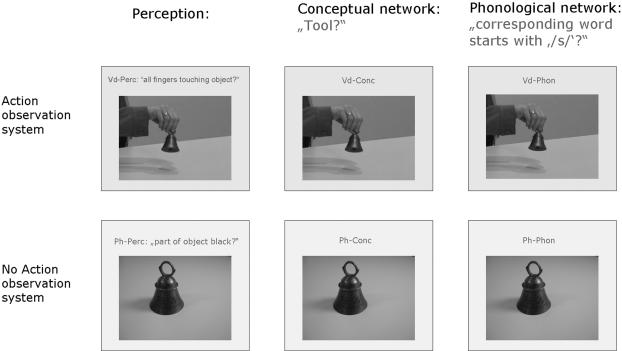
Schematic overview on the experimental design. Identical photo and video stimuli were employed across perceptual tasks (action observation/object perception), conceptual and phonological tasks. Thus differential functional imaging activations were not attributable to stimulus differences.

30 manipulable objects of everyday life were presented either as a picture or in a video showing also a hand manipulating the object in a typical way (e.g. photo of a bell – video of a hand ringing a bell). These two sets of stimuli (video, picture) were crossed with three tasks (see Figure 1) that the subjects were asked to perform:

*Perceptual task*: when videos were presented, subjects were asked to respond whether all five fingers of the hand manipulating the object touched it (Vd-Perc); when pictures were presented, subjects were asked to respond whether any part of the object was of black color (Pict-Perc)

*Conceptual task*: while watching videos (Vd-conc) and pictures (Pict-Conc), subjects were asked to respond whether the object presented was a tool (as typically used for craftsmanship).

*Phonological task*: while watching videos (Vd-Phon) and pictures (Pict-Phon), subjects were asked to respond whether the object's name started with “/s/” or not.

An overview on the experimental design is given in Fig. 1. Subjects were asked to respond by using the index and middle finger of the right hand to press keys of a MRI-compatible response device. For all tasks, in 30% of the stimuli the correct answer was yes.

Prior to the brain imaging experiment a larger set of stimuli was shown to a separate group of 10 healthy native English speaking subjects. Participants in this pilot behavioral study were asked to evaluate whether the objects presented were tools typically used for craftsmanship and which word would best describe the object. All 10 subjects of this pilot study agreed on the object's name and whether the object was a tool or not, for the 30 stimuli chosen for the brain imaging imaging experiment.

### fMRI study

The fMRI study comprised 6 blocks for each task which were presented in counterbalanced order in a pseudorandom design. The duration of each block was 24 s. Intermixed baseline blocks lasting 20 s involved fixation of a central crosshair (rest condition). Prior to the task blocks, a short sentence presented for 1s indicated the type of task which was used in the following block, as picture and video stimuli were identical across tasks.

Functional magnetic resonance imaging (fMRI) based on the blood oxygenation level-dependent (BOLD) contrast was performed using a 3T Siemens Allegra scanner housed in the Ahmanson-Lovelace Brain Mapping Center and a standard headcoil. The fMRI runs comprised 2 dummy scans followed by 600 whole-brain scans using single-shot gradient-refocused echo-planar imaging (EPI) (TR = 2.0 s, TE = 25 ms, flip angle = 90°, 36 slices).

### Data Analysis

The fMRI Data were analysed using Statistical Parametric Mapping software (SPM2, http://www.fil.ion.ucl.ac.uk/spm/, London, UK). The dummy scans were discarded. The remaining scans were realigned and spatially normalized to standard stereotaxic space using the EPI-template of the Montreal Neurological Institute (MNI). The voxel size was 3×3×3 mm. Subsequently the normalized data were smoothed using a Gaussian kernel = 8×8×8 mm in order to improve the signal-to-noise ratio. For the following parameter estimation, an appropriate design matrix was specified using a box-car function convolved with the hemodynamic response function. Data were high-pass filtered (cutoff period 128 s) to remove low-frequency signal drifts. The voxel-by-voxel parameter estimation for the smoothed data was carried out according to the general linear model. First, single contrasts for each task and comparison across tasks, as outlined in detail below, were computed on the single subject level. These contrast images were the basis of group fMRI activations using a random effects model (one-sample t-test). The statistical threshold for all contrasts were set to p<0.05, corrected (false discovery rate, FDR); the threshold for creation of the masks used for the masking procedures was set to p<0.05, uncorrected. Only clusters with at least 20 adjacent voxels are reported.

Although the experimental design of this study is a fully factorial task (perceptual, conceptual, phonological) by stimulus (video, picture) design, the hypotheses under investigation cannot be tested with main effects and interactions, but rather with specific contrasts. Indeed, the action observation task (Vd-Perc) was the only condition which directed attention to the hand manipulating the object, whereas the remaining five tasks directed attention to the objects. The fMRI data were analyzed as follows to investigate the functional relationship between action observation system and language areas:

Areas responding to action observation were revealed by the contrast Vd-Perc - rest. To account for the decision making process which was involved in the Vd-Perc condition, we also contrasted the activations during Vd-Perc with the perceptual object (Pict-Perc) condition (Vd-Perc – Pict-Perc). The conceptual and phonological networks involved in the linguistic tasks were shown in the conditions (Pict-Conc – rest) and (Pict-Phon– rest). The comparison of linguistic networks with the action observation network, was carried out in two steps: the extent of the phonological and the conceptual network relative to the action observation network was tested by a masking procedure, where the activations revealed by the contrasts (Pict-Phon– rest) and (Pict-Conc – rest) were masked exclusively by the activations revealed by the contrast Vd-Perc. The hypothesis that linguistic processing of observed actions has evolved from the fronto-parietal action observation system predicts that there would be no activated cluster for this contrast, i.e. no part of the conceptual or phonological network tested here extended beyond the action observation system. Further contrasts involving conditions with identical stimuli assessed the degree of activation between the action observation system and linguistic networks: (Vd-Perc – (Vd-Conc+Vd-Phon)) and ((Vd-Conc+Vd-Phon) – Vd-Perc). These contrasts were designed for a quantitative comparison of activity in areas involved both during linguistic processing and action perception tasks.

## Results

### Task performance

**Figure 2:**
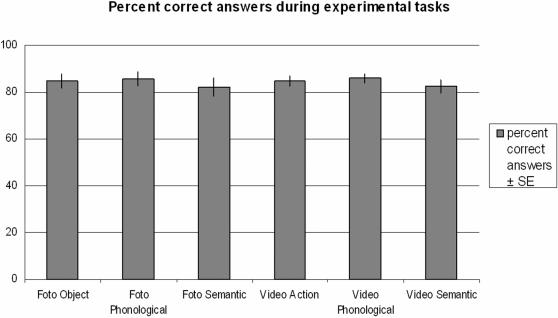
Behavioral data of correctness for all experimental tasks. There were no differences regarding correctness across tasks. However, the tasks involving video stimuli (Vd-Perc, Vd-Conc, Vd-Phon) regarded longer reaction times than the photo stimulus tasks with the video action task evoking the longest reaction times. This reflects the nature of this task, focusing attention towards the performed hand action, whereas the other tasks directed attention towards the depicted object.

The average rate of correct answers across all tasks was 84.26±1.22%. Correctness rates for the six different tasks were as following: Pict-Perc, 84.7±2.9% (S.E.), Pict-Phon, 85.6±3.1%, Pict-Conc, 82.1±3.7%, Vd-Perc 84.7±2.2%, Vd-Phon 85.9±1.9%, Vd-Conc 82.4±2.7% (Fig. 2). This rate of correct answers may be due to two factors. First, in the brain imaging experiment we used a forced-choice task, thus some of the errors were probably due to somewhat speeded responses. Second, some stimuli were also perceptually challenging; for example, only a small part of the object was black or it was not so easy to determine if all fingers or just four fingers held the object.

Pairwise comparisons of correct responses using Student's t-test (corrected for multiple comparisons) revealed no significant differences between the conditions.

Average reaction time across conditions was 1284.9±31.4 ms. Reaction times for individual conditions relative to stimulus onset ranged between 1096.2±42.4 ms and 1546.2±74.2 ms (Pict-Perc: 1103.0±68.6 ms, Pict-Phon: 1193.6±44.3 ms, Pict-Conc: 1096.2±42.4 ms, Vd-Perc: 1546.2±74.2 ms, Vd-Phon 1395.5±52.7 ms, Vd-Conc 1375.0±55.4 ms). Pairwise comparisons of reaction times revealed significantly higher reaction times for the action observation task compared to all other tasks, for the two other tasks involving video stimuli (Vd-Phon and Vd-Conc) compared to the three tasks involving photo stimuli and for Pict-Phon compared to Pict-Conc. These differences make sense. Indeed, the perceptual video task could be ‘solved’ only at the time point in the video when the hand touched the object. In contrast, the information necessary to perform the remaining tasks was present since the very first frame of the video.

### Functional imaging results

**Figure 3:**
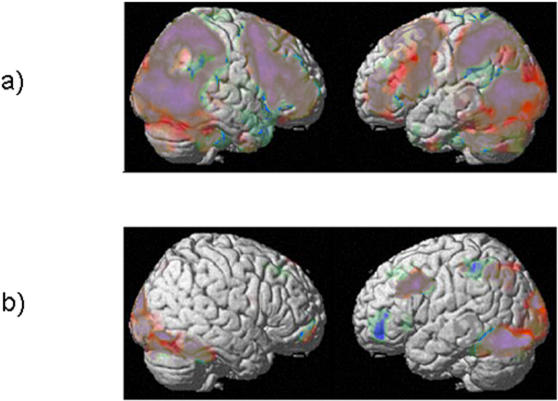
a) cortical networks activated by the decision task relating to action observation vs rest (Vd-Perc vs rest, red) and action observation vs perceptual decisions on photos of the same objects (Vd-Perc vs Ph-Perc, blue). The large bihemispheric networks found for both contrasts were very similar, suggesting that the fMRI activations found here mainly were related to action observation and not to processes of decision making or object perception required during these tasks, as well. b) Cortical networks activated during the phonological (blue) and the conceptual decision task (red) on photos of manipulable objects. The networks activated by these two linguistic tasks were entirely part of the action observation network depicted in Fig. 3a, in accordance with the hypothesis that development of language out of the mirror neuron system was driven by a process of exaptation.

As expected, the contrast Vd-Perc vs rest revealed signal increases bilaterally in exstrastriate visual regions, inferior and superior parietal regions and extensive bihemispheric frontal activations comprising premotor, inferior frontal and prefrontal (dorsolateral and ventrolateral prefrontal cortex) areas (Fig. 3a, table 1). This network is in good accordance with the bilateral network described by previous fMRI studies investigating action observation [7], [15]–[18]. The contrast Vd-Perc - Pict-Perc, which should subtract activity due to object perception and decision making, demonstrated a very similar network of bilateral parietal, premotor and prefrontal areas (Fig 3a).

**Table 1:** Peak voxel coordinates in MNI space and z-values for the fMRI contrasts revealing the action observation network.

Video Perception>rest					
region	BA	x	y	z	Z-score
right dorsal premotor	6	30	8	62	4.49
right ventral premotor	6/9	44	8	32	4.5
right inferior frontal gyrus, pars opercularis	44	46	12	22	4.49
right inferior frontal gyrus, pars orbitalis	47	34	26	−4	3.29
right dorsolateral prefrontal cortex	46	52	40	15	4.95
right ventrolateral prefrontal cortex	10	44	58	10	3.22
left dorsal premotor	6	−28	−4	56	4.32
left inferior frontal junction	9	−38	18	30	4.45
left inferior frontal gyrus, pars opercularis	44	−50	6	20	4.68
left inferior frontal gyrus, pars triangularis	45	−60	20	16	3.52
left inferior frontal gyrus, pars orbitalis	47	−50	40	−16	3.55
left pre-SMA	6	−4	16	52	3.59
right IPS	7	26	−58	50	5.21
right inferior parietal	39	32	−68	40	5.06
left precuneus	7	−30	−46	40	5.38
left anterior IPS	7	−26	−60	44	4.66
right fusiform gyrus	20	40	−44	−20	4.85
right occipital lobe	18	10	−98	16	5.58
right occipito-temporal junction	39	44	−72	10	5.21
left occipital lobe	19	−50	−58	−14	5.02
	17	10	−90	0	4.94
left cerebellum, lobule V, VI and crus I		−12	−74	−50	5.27
right cerebellum, lobule V, VI and crus I		12	−78	−50	4.63
right thalamus		26	−30	0	4.07
left thalamus		−16	−24	16	4.01

The contrast Pict-Phon vs rest revealed mainly left frontal regions including anterior inferior frontal and adjacent middle frontal gyrus, ventral premotor cortex and dorsolateral prefrontal cortex, and a left hemispheric activation of the supramarginal gyrus (Fig. 3b, table 2). The conceptual task (Pict-Conc vs rest) likewise activated a predominantly left hemispheric network including a cluster within left posterior middle frontal gyrus extending into dorsal inferior frontal gyrus and ventral premotor cortex and a parietal cluster mainly covering the angular gyrus (Fig. 3b, table 3). These areas are in good accordance with core regions described in a recent metaanalysis of fMRI studies for phonological and conceptual processes [19]. There were no significant temporal activations at the chosen threshold for both linguistic tasks, a result which is probably related to the fact that the experimental design required photos of object stimuli instead of written text or speech as basis for the linguistic tasks.

**Table 2:** Peak voxel coordinates in MNI space and z-values for the fMRI contrasts revealing the phonological network, as tested in the present study.

Photo Phonological>rest					
region	BA	x	y	z	Z-score
right ventrolateral prefrontal cortex	11	42	56	−10	
right pre-SMA	6	−4	16	52	3.23
left ventral premotor cortex	6/9	−46	6	34	4.36
left inferior frontal gyrus/middle frontal gyrus	45/46	−48	46	8	3.94
left middle frontal gyrus	9	−48	28	38	3.96
left pre-SMA	6	−6	38	44	3.75
left inferior frontal gyrus, pars orbitalis	47	−32	22	−2	3.46
left supramarginal gyrus	40	−50	−36	50	4.06
left fusiform gyrus	37	−52	−64	−16	4.85
right fusiform gyrus	20	34	−44	−22	4.58
right occipital lobe	18	14	−98	12	4.42
left occipital lobe	18	−32	−88	−18	4

**Table 3:** Peak voxel coordinates in MNI space and z-values for the fMRI contrasts revealing the conceptual network, as tested in the present study.

Photo conceptual>rest					
region	BA	x	y	z	Z-score
right ventrolateral prefrontal cortex	11	42	54	−14	3.81
left sensorimotor cortex	1/4	−50	−28	50	3.08
left inferior frontal junction	9	−50	14	34	4.11
left ventral premotor cortex	6	−42	0	38	3.38
left superior frontal gyrus	8	−4	42	58	3.8
left anterior IPS	7	−28	−50	42	3.66
left angular gyrus	39	−28	−68	36	3.48
left fusiform	37	−50	−64	−12	4.88
left occipital	18	−32	−86	−12	4.62
right occipital	18	16	−102	16	4.64

The second step of the data analysis aimed at systematic comparison of cortical networks involved in action observation and in conceptual/phonological processing in light of the hypothesis of a common evolutionary process of language networks and the mirror neuron system. Exclusive masking of the activations for the phonological network (Pict-Phon - rest) with the activation map of the action perception task (Vd-Perc - rest) did not reveal any remaining activation clusters. The corresponding masking analysis for the conceptual network (Pict-Conc – rest) revealed the same result. These results demonstrate that there were no cortical regions exclusively activated either during the conceptual task or the phonological task which were not part of the network activated by action perception.

Further statistical comparisons were related to the degree of activation during phonological or conceptual processing compared to action perception. For this comparison, the three conditions involving identical video stimuli were employed (Vd-Perc, Vd-Phon and Vd-Conc). The comparative analyses of the fMRI activations during these tasks ((Vd-Conc+Vd-Phon) vs. Vd-Perc, Vd-Conc vs Vd-Perc, Vd-Phon vs Vd-Perc) revealed no significantly higher fMRI activation for the conceptual or the phonological task than for the action perception task.

**Figure 4:**
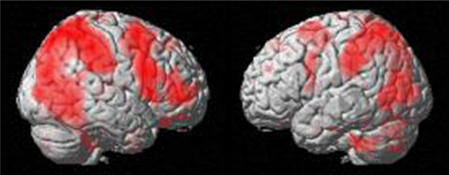
Areas showing a significantly higher activation for decision on action observation (“is the hand holding the object with all five fingers?”) than for conceptual or phonological tasks involving the same video stimuli (Vd-Perc > (Vd-Phon+Vd-Conc)). Comparison with the bilateral network for action observation (Fig. 3a) revealed that most the bilateral parietal and right frontal activations were activated when an action observation-related task was done but not if a concurrent cognitive task of conceptual or phonological decision on the same stimuli were performed.

The reverse contrast (Vd-Perc vs (Vd-Conc+Vd-Phon)) showed a widespread bilateral parietal and mainly right hemispheric frontal network of regions exhibiting higher fMRI activation during action perception than linguistic analysis, given identical video stimuli (Fig. 4, table 4). There were only small clusters in the left frontal lobe showing higher activation for action perception than for conceptual or phonological processing. Taken together with the previous analyses this result indicates a similar level of activation of the left inferior frontal cortex during action perception and linguistic tasks.

**Table 4:** Peak voxel coordinates in MNI space and z-values for the fMRI contrasts revealing the regions showing a higher activation for the action observation task than for the two linguistic tasks.

Video Perception>(Video Phonology+Video Conceptual)					
region	BA	x	y	z	Z-score
right dorsal premotor	6	26	8	64	4.81
right ventral premotor	6	38	−2	48	4.31
right inferior frontal gyrus, pars orbicularis	44	58	16	14	4.39
right inferior frontal gyrus, pars triangularis	45	58	18	6	4.98
right middle frontal gyrus	46	42	14	24	5.34
right ventrolateral prefrontal cortex	10	36	62	12	3.46
right pre-SMA	6	6	12	52	3.27
left dorsal premotor	6	−28	−4	50	3.81
left ventral premotor cortex	6	−56	6	42	4.2
left inferior frontal gyrus, pars opercularis	44	−52	4	12	3.1
left dorsolateral prefrontal cortex	46	−42	46	28	3
right superior parietal	7	18	−74	58	5.32
right IPS	7	24	−60	48	5.35
right angular gyrus	39	44	−76	26	4.83
left superior parietal	7	−22	−60	64	4.79
left supramarginal gyrus	40	−66	−32	32	4.23
left anterior IPS	40	−48	−46	52	3.74
right occipito-temporal junction	19/37	50	−62	−10	3.69
left occipito-temporal junction	19	−48	−74	−16	2.7
left cerebellum, crus I and II		−40	−48	−50	3.66
right thalamus		16	−24	12	3.88
left thalamus		−10	−16	12	3.59

The video stimuli used for the latter contrast were identical, thus the differential activation was related to differential task instructions and not to stimulus perception.

## Discussion

The experiment reported here adopted a design that required subjects to process identical visual stimuli while performing different tasks: an action perception task, and two ‘linguistic’ tasks, a phonological task and a conceptual task. With this experimental design, it is possible to test whether the overt linguistic processing of observed object-oriented action recruits cortical areas not engaged by action perception, and/or activates fronto-parietal action perception areas to a higher degree. Both results would support the hypothesis of some independence of linguistic processing of visual stimuli with respect to fronto-parietal areas concerned with action perception. However, we did not find any area specifically activated during the two linguistic tasks, and we did not find any area with higher activity during the linguistic tasks. We argue that these results are more readily compatible with the hypothesis that language –as far as linguistic processing of visual stimuli is concerned, at the very least – evolved by co-opting fronto-parietal systems concerned with action perception, a process known as exaptation.

Although previous studies have reported shared activation for action (or action observation) and language [8], [17]- activations typically interpreted in support of the embodied semantics framework [20], [21]- those previous studies could not comment on differential activations between action and language, since their stimuli widely differed between action and language tasks. Thus, the main novel finding of our study is that linguistic processing of visual stimuli related to actions occurs within a subset of fronto-parietal areas concerned with action perception.

It could be argued that action perception entails automatic linguistic processing, and that the left inferior frontal areas not differentially activated during action perception and the linguistic tasks are indeed exclusively linguistic in nature. While this argument is logically correct, it is unlikely to be true. Indeed, our data show that the inferior frontal cortex also has higher activity during action perception (Vd-Perc) compared to object perception (Pict-Perc), two tasks ostensibly very similar with regard to possible automatic linguistic processing, but dissimilar with regard to action perception itself. Thus, the deflationary explanation that invokes automatic linguistic processing in left inferior frontal cortex in all tasks does not easily account for all experimental results presented here. Furthermore, a virtual lesion study using repetitive TMS has shown that a transient disruption of neural activity in the left (and right) inferior frontal cortex results in imitation deficits, but not in more general visuo-motor deficits [22]. This result can hardly be reconciled with a purely linguistic property of left inferior frontal cortex.

It could also be argued that the increased signal in fronto-parietal areas is only due to the increased attentional demands of the action perception task, given the increased RT for this task. It should be noted, however, that the increase in reaction time for this task was very small in relation to the overall duration of each task block during scanning. Therefore, we consider it unlikely that the difference in reaction time across tasks was of substantial influence on the fMRI activations. Moreover, the “attention” argument cannot account for the lack of increased signal in the left inferior frontal cortex during action perception, compared to the linguistic tasks. The selectivity of the effect argues against a non specific attention effect.

Our design also allowed us to compare activity during action perception and during perception of static pictures that comprised all the visual elements of the action stimuli. Thus, this comparison reveals brain activity that is quite specific to action observation, rather than to the complex visual elements that invariably go together with observed actions. This comparison in our experiment shows robust bilateral activation in fronto-parietal areas, revealing that this large network is indeed specifically concerned with action perception. Thus, the result of our specific contrast support the ‘mirror neuron’ interpretation of the vast number of previously published papers showing similar fronto-parietal activations in a variety of experimental conditions [7], [18], [23]–[26].

Fronto-parietal areas concerned with action perception are bilateral, whereas our linguistic tasks recruited exclusively left hemisphere areas. This shift from bilateral activity for action perception to a predominantly left lateralized language system may have been favored by a lateralization, in humans, of ‘mirroring’ responses to action sounds, as shown by single pulse TMS [27] and fMRI [28].

To conclude, when visual stimuli concerning object-oriented actions are processed perceptually, they activate a large bilateral fronto-parietal network. When the same stimuli are processed linguistically, they activate only a subset of this network and no additional areas. This pattern of activity supports the evolutionary hypothesis that neural mechanisms for language in humans co-opted phylogenetically older fronto-parietal neurons concerned with action perception, such as mirror neurons in macaques.
